# A conspectus on the Canacidae (Diptera) of Brazil

**DOI:** 10.3897/zookeys.162.2370

**Published:** 2012-01-05

**Authors:** Wayne N. Mathis, Luciane Marinoni

**Affiliations:** 1 Department of Entomology, NHB 169, PO Box 37012, Smithsonian Institution, Washington, D. C. 20013-7012, USA; 2 Curadora da Coleção Entomológica Pe. Jesus Santiago Moure, Departamento de Zoologia, Universidade Federal do Paraná, Jardim das Américas, 81531-980 - Curitiba, Paraná, Brazil

**Keywords:** Diptera, Canacidae, conspectus, new species, Brazil

## Abstract

Species of Canacidae *sensu lato* of Brazil are reviewed, including the subfamilies Canacinae and Tethininae. Included are seven species in five genera with two species, *Nocticanace austra* and *Nocticanace packhamorum*, from southern Brazil being newly described. To facilitate identification, we have included keys and diagnose to taxa at all levels.

## Introduction

The Canacidae of Brazil have never been treated comprehensively even though specimens are often abundant and species are relatively diverse on beaches of this large Neotropical and biologically diverse country where life on the beach is often a way of life. This deficiency is not uncommon, however, and characterizes many insect families occurring on beaches, especially groups that have relatively few species, that are collected infrequently despite being common locally, and that have no species of known economic importance. Although the Canacidae lack pestiferous species, study of the family is warranted, as its species comprise an important component of the beach fauna. Beyond satisfying the immediate objective--a taxonomic conspectus of the Brazilian fauna--we are also seeking to discover and contribute toward other aspects of their natural history, such as their distribution, historical biogeography, ecology, behavior, and biodiversity. The underlying basis for all of these studies, however, is the taxonomy of the included taxa.

True flies of the family Canacidae occur in cool-temperate and tropical zones of the world, primarily on or near seashores with oceanic climates. A few species are found inland, usually in saline or alkaline environments, but occasionally in meadow-like habitats or in freshwater, such as the streams of Hawaii. Worldwide there are 308 species in the family (6 subfamilies, 27 genera) (Munari and Mathis 2010; Munari and Stuke 2011), and from Brazil, we now have records of seven species and others are likely to be discovered here. The species of Brazil are in two subfamilies and five genera. Although the Canacidae of Brazil have never been treated comprehensively until now, the research published here had its genesis and basis in the works of others, as noted in the synonymy for the taxonomic categories from family to species that are included here. The classification adopted here is intended to provide perspective for this faunistic study and to serve as the organizational structure for this paper.

The historical record concerning Canacidae from Brazil began slightly more than a century ago when Williston (1896) described two species (*Anthomyza cinerea* (= *Tethina willistoni* (Melander)) and *Rhicnoessa xanthopoda*) from specimens collected on the island of St. Vincent (Caribbean). During the intervening 100 years, another species, *Tethina albula* (Loew), had been reported from Brazil (Melander 1952; Mathis and Munari 1996) but was apparently based on a misidentification. We have not examined any specimens of *Tethina albula*, and all specimens that are light colored, including mostly pale setae, are *Tethina willistoni*. Herein we review seven species in five genera that occur in Brazil. Three of these genera are reported for the first time from Brazil, as are four of the species. Two species, *Nocticanace austra* and *Nocticanace packhamorum*, are new to science and are described in this paper.

Because many species of Canacidae are widespread, especially those that occur in coastal marine habitats, we have examined most New World species, including primary types, to determine the correct identifications and valid names for the included species.

## Materials and methods

The descriptive terminology for external structures and many internal structures follows that published in the Manual of Nearctic Diptera (J. F. McAlpine 1981). For structures of the male terminalia, however, we have adopted the terminology that Cumming et al. (1995) have suggested. Because specimens are small, usually less than 5.0 mm in length, study and illustration required use of dissecting and compound microscopes. Two wing ratios used in the descriptions are defined below (ratios are averages of three specimens: the largest, smallest, and one other).

1. Costal section ratios are the relative straight line distances between the apices of the subcosta and vein R_1_: apices of R_1_ and R_2+3_; and apices R_2+3_ and R_4+5_.

2. M vein ratio: the straight line distance along M between crossveins (r-m and dm-cu)/distance apicad of crossvein dm-cu.

Label data from each specimen were recorded and listed alphabetically according to country, state or province, county, and specific locality, such as city. As available, date of collection, collector, sex, and specimen location were listed. Label data from holotype specimens were recorded exactly, and clarifying information, such as script style and label color, is enclosed within brackets.

Dissections of male and female genitalia and descriptions were performed using the method of Clausen and Cook (1971) and Grimaldi (1987). Microforceps were used to remove abdomens, which were macerated in a potassium or sodium hydroxide solution. Cleared genitalia were rinsed in distilled water and 70% ethanol and then transferred to glycerin for observation. If necessary for proper orientation, the genitalia were transferred from glycerin to glycerin jelly. The glycerin jelly was heated, and the genitalia appropriately oriented. After cooling, the embedded specimen became immobilized. Abdomens were placed in an attached plastic microvial filled with glycerin and attached to the pin supporting the remainder of the insect from which it was removed. For freshly caught specimens, we recommend that the epandrium and associated structures of the male terminalia be teased open, thus allowing examination of these structures and identification of the species without need of dissection.

Species’ descriptions are composite and not based solely on the holotypes, and paired structures are described in the singular except where the context makes this inappropriate.

Although most specimens for this study are in the Department of Zoology, Universidade Federal do Paraná, Curitiba, Brazil (DZUP) and the National Museum of Natural History, Smithsonian Institution, Washington, D. C. (USNM), we also studied numerous specimens that were borrowed and are deposited elsewhere. These include (acronyms that are used in the text are noted first):

BPBM Bernice P. Bishop Museum, Honolulu, Hawaii, USA

BMNH The Natural History Museum (former British Museum (Natural History)), London, United Kingdom

FIOC Fundação Instituto Oswaldo Cruz, Rio de Janeiro, Brazil

MCZ Museum of Comparative Zoology, Harvard University, Cambridge, Massachusetts, USA

MZUSP Museu de Zoologia da Universidade de São Paulo, São Paulo, Brazil

TAU Tel Aviv University, Tel Aviv, Israel

## Systematics

### Family Canacidae

Jones

http://species-id.net/wiki/Canacidae

Canacenae Jones 1906: 170, 198 [as a subfamily of Ephydridae, incorrect formation of the family-group name]. Type genus: *Canace* Haliday 1837.
Canaceidae . Hendel 1916: 297 [incorrect formation of the family-group name]. Wirth 1951: 245–275 [revision]; 1975: 1–5 [Neotropical catalog]; 1987: 1079–1083 [North American manual].
Canacidae . Enderlein 1935: 235. Mathis 1982: 1–29 [classification]. Buck 2006: 391–392 [familial status]. Munari and Mathis 2010: 1–84 [world catalog].
Tethinidae Hendel 1916: 297; 1917: 45. Type genus: *Tethina* Haliday. Foster 1976b: 1–4 [Neotropical catalog]. Mathis and Munari 1996: 1–27 [world catalog]. McAlpine 2007: 42 [synonymy].

#### Diagnosis.

 The family Canacidae, *sensu lato*, is distinguished from other families of the Carnoidea by the following combination of characters: Exclusively or tending to occur in saline habitats (secondarily in freshwater habitats). Minute to moderately small flies, length 0.91–5.0 mm. *Head:* Postocellar setae developed (absent or reduced in some Canacinae); dorsal fronto-orbital seta lateroclinate; oral vibrissae weakly differentiated, except for *Dasyrhicnoessa* Hendel species. Arista dorsal. Face sometimes characterized by 2 shiny protuberances laterad to the facial cavity, just above vibrissal pore (*Tethina*, *Pseudorhicnoessa*) or nearby (*Afrotethina*, *Horaismoptera*); face strongly depressed and short (*Dasyrhicnoessa*, Horaismopterinae) or with medial carina (*Tethina*) or even distinctly convex (Canacinae). Gena bare, except for ventral or nearly ventral row of setae (peristomal setae), or even with a few anaclinate, strong setae (Canacinae). Buccal parts generally strongly sclerotized in Canacinae. *Thorax:* Precoxal bridge developed. Prescutellar acrostichal setae developed; presutural dorsocentral setae differentiated; anepisternum with 2–3 developed posterior setae, bearing enlarged, dorsally curved seta at posterodorsal corner; usually 1 katepisternal seta present; proepisternal seta developed. Wing generally hyaline, bearing fine, dense microtrichia; subcosta weakened apically, close to vein R_1_; vein A_1_ short (except in the sub-Antarctic genus *Apetaenus*); vein A_2_ long, present as a fold. *Abdomen:* Pregenital sclerites of male short and fused; male tergite 6 fused with sternite 8, forming a usually symmetrical (except in some species of *Tethina*), pregenital sclerite; male sternite 7 lost; postgonites firmly connected laterally to base of phallapodeme, distinctly anterior to basiphallus; hypandrium forming a sheath or phallic mantle around the postgonite and basiphallus; epandrium bearing 1–2 pairs of surstyli ventrally, sometimes anterior surstylus lacking (Canacinae, *Tethina*); posterior surstylus partially articulated or fused with epandrium; inner basal corner of surstylus connected to broad interparameral sclerite; cercus very short to exceptionally developed (Horaismopterinae); postabdomen of female more or less telescopically retractile; 2 sclerotized spermathecae variable in shape, below with a narrower cylindrical extension into the spermathecal duct; cercus subcylindrical to compressed, or even tapered distally, sometimes bearing stout to pointed, spinelike setulae.

#### Discussion.

 Our concept of Canacidae includes what had been considered as two families, the Canacidae and Tethinidae. At the familial level, J. F. McAlpine (1989: 1472) identified five synapomorphies that link Canacidae with Tethinidae and noted that “...these are clear indications of a sister-group relationship between them ... and may even indicate that they are subgroups of a single family.” Other authors (Hennig 1958; Griffiths 1972; McAlpine 1982; Freidberg 1995) have also suggested a relationship with the family Tethinidae, and Griffiths (1972) further noted some affinities with the Chloropidae and Milichiidae. According to J. F. McAlpine’s (1989) cladogram, which included an analysis of 25 characters for the families Canacidae and Tethinidae, the superfamily Carnoidea (= Chloropoidea) comprises the families with the following relationships in parenthetic notation: ((Australimyzidae, Braulidae) Carnidae)((Tethinidae, Canacidae)((Milichiidae, Risidae) ((Cryptochetidae, Chloropidae)))).

More recently, Buck (2006) and D. K. McAlpine (2007) provided rather compelling character evidence, substantiating that these two families are closely associated, and more specifically that the Canacidae *sensu stricto* are an included lineage within the Tethinidae. Thus, not to include the Canacidae within the Tethinidae would render the Tethinidae as a paraphyletic family. Buck and D. K. McAlpine cited ten synapomorphies that corroborate the monophyly of the family Canacidae *sensu lato* (the family-group name Canacidae is older than Tethinidae). These synapomorphies are (only derived state cited): (1) Precoxal bridge present; (2) anepisternum with enlarged, dorsally curved setae at posteroventral corner; (3) vein A_2_ long, present as a fold; (4) male sternite 6 reduced and divided medially; (5) male tergite 6 fused with sternite 8, forming a symmetrical pregenital sclerite; (6) male sternite 7 lost; (7) postgonites firmly connected laterally to base of phallapodeme, distinctly anterior to basiphallus; (8) hypandrium forming a sheath or phallic mantle around the postgonite and basiphallus; (9) cuticle of larva with covering of fine spicules, and (10) halobiontic in habitat preference, secondarily in freshwater habitats. Buck (2006) further suggested that the sister group to Canacinae *sensu stricto* is the subfamily Apetaeninae and not Zaleinae and provided four characters as corroborative evidence for this relationship: (1) antennae broadly separated, inserted more or less on protuberant facial tubercles; (2) clypeus distinctly enlarged and produced anteriorly; (3) prementum distinctly emarginated apically; and (4) tentorial arms of head capsule enormously developed and strongly sclerotized.

#### Key to Subfamilies of Canacidae sensu lato from Brazil

**Table:** 

1	Frontal orbit with 3–5 major lateroclinate setae, foremost near level of ptilinal fissure, in addition to inner series of 3 or more proclinate-inclinate, shorter setae or setulae; proclinate-inclinate interfrontal setae in 2 distinct series; pair of convergent, often widely spaced, postocellar setae present; if absent then wing with distinct, black spots (*Tethina lusitanica*); costa along marginal cell with a continuous series of closely placed, short, black, anterior spinules, and no series of longer, widely spaced spines; discal and second basal cells separate; anal cell closed; vein A_1_+CuA_2_ (6th longitudinal) not extending distinctly beyond anal cell, even as a sharp fold in membrane	Tethininae
–	Fronto-orbital setae not arranged as above; if biseriate interfrontal setae present, then either convergent postocellar setae absent or anal cell open distally; other characters variable	2
2	Wing either vestigial, or with long vein A_1_+CuA_2_ extended to margin; fronto-orbital setae normally 3, of which middle one is reclinate and further from eye than others; female: syntergite 1+2 longer than rest of abdomen; endemic in the subantarctic archipelagos	Apetaeninae (not yet known from South America)
–	Wing normally developed, with vein A_1_+CuA_2 _scarcely extended beyond anal cell; if 3 fronto-orbital setae present, then middle one not farther from eye than others; syntergite 1+2 at most as long as or normally shorter than rest of abdomen; not inhabiting the subantarctic archipelagos	Canacinae

#### Subfamily Canacinae

http://species-id.net/wiki/Canacinae

Canaceinae . Hendel 1913: 93 [as a subfamily of Ephydridae, incorrect formation of the subfamily-group name].
Canacinae . Enderlein 1914: 326 [as a subfamily of Ephydridae]. Malloch 1933: 4 [as a subfamily of Ephydridae]. Mathis 1982: 2 [as a subfamily of Canacidae, phylogeny]. McAlpine 2007: 43 [review, diagnosis, status]. Munari and Mathis 2010: 11–27 [world catalog].

##### Diagnosis.

 Adult. Minute to moderately large surf flies, body length 1.60–5.00 mm; blackish, brownish, yellowish, or gray, often invested with whitish to grayish microtomentum. *Head:* Antennae broadly separated, inserted more or less on protuberant facial tubercles; subcranial cavity large; 3–5 lateroclinate fronto-orbital setae. Face slightly convex to concave; setae usually sparse except for mesoclinate vibrissal seta; vibrissal angle unmodified; clypeus prominent, enlarged, wide. Gena high, bearing 1–4 dorsoclinate genal setae. Subcranial cavity enlarged; labella short, nongeniculate; prementum short, broad, deeply incised distally, distinctly emarginated apically; tentorial arms of head capsule enormously developed and strongly sclerotized. *Thorax:* Mesonotum with 4 or more dorsocentral setae. Wing usually hyaline; C extended to M and with subcostal break only; Sc complete and separate from R_1_ almost to its apex; cells br, bm, dm, and cup complete; A_1_ short. Precoxal bridge present. *Abdomen:* Male tergites 1–6 exposed; spiracles 1–6 in posteroventral portion of tergite, spiracle 7 also in tergite 6; terminalia symmetrical; surstylus fused with epandrium; hypandrium usually with lateral arms extended above aedeagus, fused into posteriorly directed process; aedeagus relatively short; cercus usually weak. Female cerci well sclerotized, long, approximate, bearing a strong apical seta, sometimes preceded by similar but smaller setae; ventral wall of genital chamber with V- or ring-shaped sclerite; spermathecae 2.

Egg. Simple, ovoid; with microscopic reticulations.

Third-instar larval length 5–6 mm; tapered anteriorly and posteriorly from about 4th abdominal segment and terminated posteriorly in a slender retractable respiratory tube. Abdominal segments 2–7 with creeping welts. Prothoracic spiracle a slender retractable filament. Posterior spiracles with 3 oval spiracular openings arranged with longitudinal axis at slightly less than right angles to adjacent opening; each spiracular plate with 4 tufts of interspiracular setae. Cephalopharyngeal skeleton with ventral cornu truncate, appearing broken at apical margin; mandibles approximate anteriorly, separated posteriorly by small V-shaped accessory oral sclerite; anterior ventrolateral extensions of tentoropharyngeal sclerite narrowly fused with ventral bridge of hypopharynx; parastomal bars prominent, united by a thin fenestrated epipharyngeal sclerite.

Puparium. Brown, similar in size and form to third-instar larva, rather spindle-shaped, curved at each end; integumental spinules more prominent than on larva and anterior respiratory processes fully extended.

##### Biology.

 All Canacinae from the New World occur in intertidal habitats and are sometimes called surf flies. Although the natural history of the subfamily is poorly known, the larvae and adults are probably grazers on algae or are saprophytic in both saline and freshwater habitats. In Brazil, all species of the subfamily Canacinae occur in the littoral biotic region.

##### Discussion.

 Adult of Canacinae are similar and sometimes confused with shore flies (Ephydridae) and most species described in the 19^th^ century were placed in the Ephydridae. Canacids are distinguished by the wing venation (cells bm and cup complete) and by the additional abdominal segments (5 in ephydrid males, 6 in canacids), which in females terminate as an elongate and fused epiproct+cercus that bears enlarged, apical setae.

The Canacinae now include 122 valid species that are placed in 11 genera (Wirth 1951; Mathis 1992; Munari and Mathis 2010). The New World fauna comprises five genera and 35 species (Wirth 1965, 1975, 1987; Mathis 1992). No fossils are known. Mathis’ catalog (1992) included all species then known plus references to papers containing keys and illustrations. The recent catalog of Munari and Mathis (2010) is a complete updating, including keys to all known genera. In the New World, Mathis (1989, 1997) reviewed the surf-fly fauna for the Caribbean and Gulf of Mexico.

Mathis (1982) proposed a classification for the Canacinae *sensu stricto* that should be revised. The subfamily includes two tribes, Canacini and Nocticanacini. The Canacini are represented in the New World by a single genus, *Canacea* Cresson, which belongs to the subtribe Dynomiellina. The Nocticanacini are represented by three genera in the New World, *Canaceoides* Cresson, *Nocticanace*, and *Paracanace*. *Procanace*, the fifth New World genus, was initially placed in Nocticanacini, but it is now evident that this genus is the sister group to all other genera of the subfamily Canacinae.

##### Key to Genera of Canacinae from Brazil

**Table:** 

1	Interfrontal setae absent, although anterior 1/3 of frons occasionally with scattered setulae	*Procanace* Hendel
-	Interfrontal setae present, 1 or more pairs in additional to any setulae	2
2	One interfrontal seta present; postocellar setae either much reduced or lacking	*Nocticanace* Malloch
-	Two interfrontal setae present; postocellar setae well developed, proclinate and slightly divergent	*Paracanace* Mathis and Wirth

##### Genus Nocticanace

Malloch (35 species worldwide; 2 from Brazil)

http://species-id.net/wiki/Nocticanace

Nocticanace Malloch 1933: 4. Type species: *Nocticanace peculiaris* Malloch, by original designation. Wirth 1951: 269–274 [revision]; 1975: 2–3 [Neotropical catalog]. Munari and Mathis 2010: 20–24 [world catalog].

###### Diagnosis.

 Small to medium-sized beach flies, body length 1.80–3.70 mm; general coloration grayish black to black. *Head:* Interfrontal setae 1 pair; postocellar setae either absent or much reduced, less than 1/4 length of ocellar setae; ocelli arranged to form an isosceles triangle, distance between posterior ocelli greater than that between either posterior ocellus and the anterior ocellus. Two-3 long dorsoclinate genal setae; anteroclinate genal setae moderately well developed, at least 1/2 length of larger dorsoclinate genal setae. Epistomal margin sinuous; clypeus low, width subequal to length of antenna. Palpus grayish black, bearing 1 to several long setae, each seta 2–3 times greatest width of palpus. *Thorax:* Anepisternum with scattered setulae; proepisternal seta absent; katepisternal seta present, well developed. Legs entirely dark colored, grayish black; forefemur bearing 4–6 long and evenly spaced setae along posteroventral margin, length of setae at least equal to and usually greater than width of femur.

###### Discussion.

 This is the most species-rich genus of surf flies (Canacinae; 35 species) and has greatest species diversity in the Old World (Mathis 1992). The New World fauna now comprises 14 species. The species known from Brazil belong to the *pacifica*, and *galapagensis* groups.

###### Annotated Key to Species Groups of the Genus Nocticanace

**Table:** 

1	Anterior notopleural seta absent	2
–	Anterior notopleural seta present	3
2	Apical scutellar setae distinctly dorsoclinate	the *pacifica* group [20 species; New World (Brazil) and Old World (Pacific and Indian Oceans, especially Oceania)]
–	Apical scutellar setae straight to very slightly curved dorsally	the *texensis* group [4 species; Caribbean, Gulf of Mexico and southeastern United States; revised by Mathis 1989: 594–599]
3	Length of apical section of vein CuA_1_ twice or more length of crossvein dm-cu	the *galapagensis* group [9 species; Galápagos Islands, Brazil (Paraná, São Paulo), and southwestern North America]
–	Length of apical section of vein CuA_1_ subequal to length of crossvein dm-cu	4
4	Apical scutellar setae distinctly dorsoclinate	the *ashlocki* group [1 species, *Nocticanace ashlocki* Wirth; Galápagos Islands]
–	Apical scutellar setae not dorsoclinate	the *chilensis* group [1 species, *Nocticanace chilensis* (Cresson); Chile (there are numerous undescribed species in this group)]

###### The pacifica Group

**Diagnosis.** Coloration generally dark, grayish brown to grayish black but with exceptions (*Nocticanace flavipalpis* and *Nocticanace litorea*: lighter, with some tan coloration on the body and legs extensively yellowish). *Head:* 2 large, dorsoclinate, genal setae. *Thorax:* Acrostichal setulae absent; apical scutellar setae distinctly dorsoclinate; anterior notopleural seta absent; proepisternal seta(e) present; anepisternum with scattered setulae; katepisternal seta present. Legs usually entirely dark, grayish brown to black (*Nocticanace flavilpalpis* and *Nocticanace littorea* are exceptions with yellowish legs); forefemur with 4–6 long and evenly spaced setae along posteroventral margin, length greater than width of femur; midfemur of male lacking a comblike row of setae; hindtibia lacking spinelike setae apically. Wing with length of apical section of vein CuA_1_ long, about twice length of crossvein dm-cu; vein M index 0.44.

####### 

######## Nocticanace packhamorum

Mathis & Marinoni sp. n.

urn:lsid:zoobank.org:act:47F40DBB-80C5-4D20-B07D-256F39CFA758

http://species-id.net/wiki/Nocticanace_packhamorum

Figs 1–2

######### Diagnosis.

**Figures 1–2.**
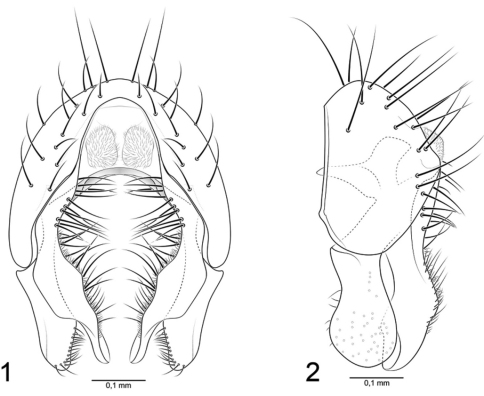
*Nocticanace packhamorum* **1** epandrium, posterior view **2** same, left lateral view.

 As in species group diagnosis with the following additions: Small to moderately small beach flies, body length 1.85–2.45 mm, of the *pacifica* group (see key to species groups). *Head:* Coloration of face and gena lighter, mostly whitish gray. Palpus yellowish gray to gray. *Thorax:* Brown coloration of mesonotum extended laterally and ventrally to about dorsum of notopleuron, thereafter gradually becoming more whitish gray with some very faint greenish tinges. Pleural areas mostly whitish gray. Legs concolorous, mostly gray to blackish gray; dorsum of femur and to a lesser extent tibia somewhat microtomentose, lightly grayish; tarsi black. *Abdomen:* Dorsum mostly grayish; median portion of each tergite with some brownish-purplish coloration, lateral margins often faintly bluish gray. Male terminalia (Figs 1–2): Surstylus deeply cleft ventrally, with a distinct anterior and posterior lobe; anterior lobe moderately slender and long, in lateral view with posterior margin angulate, moderately rounded apically, in posterior with medial surface bearing numerous, prominent, setulae along most of margin, medial portion in posterior view rectangular, apical 1/3 abruptly narrowed; posterior lobe in posterior view narrowed sub-basally, thereafter ventrally slightly expanded to form a broadly rounded apex, in posterior view with short setulae along medial surface apically.

######### Type material.

 The holotype male is labeled **“BRAZIL.** S[anta]. Catarina: Barra Velha (26°38'S, 48°40.9'W; beach), 29 Apr 2010[,] D. & W. N. Mathis/USNM ENT 00118070 [plastic bar code label]/HOLOTYPE ♂ *Nocticanace packhamorum* Mathis & Marinoni, DZUP [red].**”**The holotype is double mounted (minuten in a block of plastic), is in excellent condition, and is deposited in DZUP. Seventeen paratypes (13♂, 4♀; DZUP, USNM) bear the same label data as the holotype.

######### Distribution.

*Neotropical:* Brazil (Santa Catarina).

######### Etymology.

 The specific epithet, *packhamorum*, is a Latin genitive patronym to recognize and honor Dean and Ieda Packham, who guided us to the type locality and offered hospitality.

######### Remarks.

 Finding a species of the *pacifica* group along the Atlantic beaches of southern Brazil was unanticipated.

###### The galapagensis Group

**Diagnosis.** *Thorax:* Acrostichal setae lacking; apical scutellar setae nearly straight in lateral view, slightly convergent in dorsal view, but not distinctly curved dorsally; anterior notopleural seta present but weaker than posterior seta; proepisternal seta(e) present; midfemur of male lacking comblike row of setae; hind basitarsomere lacking spinelike basoventral setae. Wing with length of apical section of vein CuA_1_ long, length nearly twice that of crossvein dm-cu; M vein index 0.42–0.49.

**Discussion.** The *galapagensis* group now comprises nine species with the addition of the new species described below. Previously, there were eight species (*Nocticanace arnaudi* Wirth, *Nocticanace cancer* Wirth, *Nocticanace curioi* Wirth, *Nocticanace darwini* Wirth, *Nocticanace galapagensis* (Curran), *Nocticanace scapanius* Wirth, *Nocticanace spinicosta* Wirth, and *Nocticanace usingeri* Wirth) that were only known from the Galápagos Archipelago and southwestern Nearctic Region. The discovery of *Nocticanace austra* from southern Brazil is a major range extension for this species group and perhaps indicates a more extensive distribution in southern South America for the group. Better sampling in southern South America is urgently needed to test this possibility.

####### 

######## Nocticanace austra

Mathis & Marinoni sp. n.

urn:lsid:zoobank.org:act:8852F673-4335-4CB4-B99A-A53F9E0F250B

http://species-id.net/wiki/Nocticanace_austra

Figs 3–6

######### Diagnosis.

**Figures 3–6.**
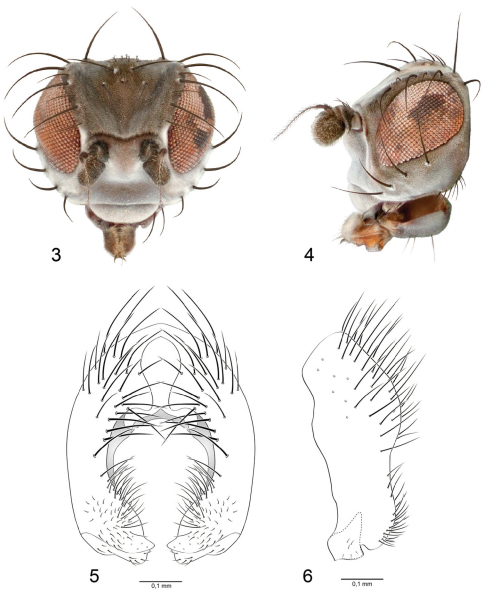
*Nocticanace austra* **3** head, anterior view **4** same, lateral view **5** epandrium, posterior view **6** same, left lateral view.

 As in the species group diagnosis with the following additions: Small to moderately small beach flies, body length 1.80–2.40 mm, of the *galapagensis* group (see key to species groups). *Head* (Figs 3–4): 3 large dorsoclinate and 1 inclinate genal setae. *Thorax:* Scutellar disc with 1 pair of setae, apical scutellar setae very shallowly curved, not distinctly oriented dorsally compared with lateral scutellar setae. Legs generally gray, with basitarsomeres blackish gray dorsally. *Abdomen:* Tergites generally gray or slightly brownish gray medially. Male terminalia as follows (Figs 5–6): Epandrium in posterior view bearing long setulae on dorsal half, with medial projection at level of dorsal 1/3 from each lateral arm, forming a cercal cavity, but cerci not evident; medial margin thereafter ventrally forming a wide cavity that narrows ventrally because of medially directly surstyli; surstylus broadly attached or fused to ventral margin of epandrium, in lateral view only slightly narrower than ventral portion of epandrium, essentially an extension of epandrium, slightly swollen posteroventrally, bearing numerous short setulae along posterior margin, ventral margin shallowly bifurcate, forming posterior and anterior lobes, posterior lobe slightly shorter than anterior lobe, gently rounded; anterior lobe more robustly developed than posterior lobe, bluntly rounded to truncate apically, very slightly produced anteroventrally as a shallow, obtuse, point, in posterior view with posterior lobe of surstylus extended medially, pointed apically, anterior lobe more broadly developed apically.

######### Type material.

 The holotype male is labeled **“BRAZIL.** São Paulo: Praia do Estaleiro (23°20.5'S, 44°53'W; beach), 30Mar2010[,] D. & W. N. Mathis/USNM ENT 00118071 [plastic bar code label]/HOLOTYPE ♂ *Nocticanace austra* Mathis & Marinoni, DZUP [red].**”** The holotype is double mounted (minuten in a block of plastic), is in excellent condition, and is deposited in DZUP. Five paratypes (4♂, 1♀; DZUP, USNM) bear the same label data as the holotype.

Other Specimens examined from Brazil. *PARANÁ.* Matinhos (N.; 25°46.4'S, 48°30.8'W; 3 m; beach/estuary), 9 Apr 2010, D. and W. N. Mathis (1♂; USNM); Paranaguá (Rio Itiberê; 25°31.4'S, 48°30.3'W; 3 m), 23 Jan 2010, D. and W. N. Mathis (1♀; DZUP).

######### Distribution.

*Neotropical:* Brazil (Paraná, São Paulo).

**Etymology.** The specific epithet, *austra*, is of Latin derivation and means southern, referring to the distribution of this species in the Southern Hemisphere.

######### Remarks.

 This species differs from congeners in the *galapagos* group in structures of the male terminalia, especially the shape of the surstylus (see figures and description above). The surstylus has a shallow, ventral bifurcation, somewhat like *Nocticanace wirthi*, but is more narrowly developed, like *Nocticanace panamensis*. The anteroventral surstylar lobe is slightly longer than the posterior lobe.

##### Genus Paracanace

Mathis and Wirth (8 species in the New World; 1 from Brazil)

http://species-id.net/wiki/Paracanace

Paracanace Mathis and Wirth 1978: 524. Type species: *Paracanace hoguei*Mathis and Wirth 1978, by original designation. Mathis 1989: 600–603 [review of Caribbean and nearby fauna]; 1992: 10 [world catalog]; 1997: 140–148 [review of *hoguei* group]. Munari and Mathis 2010: 24–24 [world catalog].
Canace , in part, of authors. Wirth 1975: 1 [Neotropical catalog].

###### Diagnosis.

 Small to moderately small beach flies, body length 1.40–2.60 mm; generally densely microtomentose, gray, with face and gena usually whitish gray, frons light brown, mesonotum with some brown coloration. *Head:* Interfrontal setae 2; postocellar seta well developed, proclinate and very slightly divergent, subequal in length to interfrontal setae; ocelli arranged in isosceles triangle, with greater distance between posterior ocelli. Two to 3 large dorsoclinate genal setae; anteroclinate genal seta well developed, subequal in length to larger dorsoclinate genal setae; epistomal margin sinuous; clypeus low, width more than 4× height; palpus yellowish. *Thorax:* Mesonotum darker than pleural areas, usually light to blackish brown, becoming lighter laterally. Acrostichal setulae in 2–4 irregular rows, with a distinctly larger prescutellar pair; scutellar disc lacking setulae; apical scutellar setae not oriented dorsally; anterior notopleural seta usually present (very weak or absent in one species); proepisternal seta(e) present; anepisternum with scattered setulae; katepisternal seta present. Femora and tibiae gray to blackish gray; tarsomeres yellow to dark brown, apical 2–3 tarsomeres darker; midfemur of male bearing comblike row of setae along posteroventral surface; midtibia bearing short evenly spaced setulae along ventral surface; hindtibia lacking spinelike setae apically. Wing with length of apical section of vein CuA_1_ twice or more that of crossvein dm-cu; M vein ratio 0.35–0.45. *Abdomen:* Male terminalia: Surstylus a simple, narrow, posteriorly shallowly curved, setulose process extended from ventral margin of epandrium.

###### Discussion.

 Like *Canacea*, all of the described species of *Paracanace* occur in the New World, with primarily tropical or subtropical distributions (Mathis and Wirth 1978).

Although two species groups are recognized in the key to species within *Paracanace*, adhering to the cladogram for the species of this genus (Mathis and Wirth 1978: 535), these groups are mostly for convenience and no phylogenetic signal should be attributed.

###### Key to Species of Paracanace

**Table:** 

1	Costal vein between humeral crossvein and subcostal break bearing a row of long spinelike setae, setal length subequal or greater than width of 1st costal cell (the *hoguei* group)	2
–	Setae along anterior margin of wing much shorter, not more than 1/2 width of 1st costal cell (the *maritima* group)	5
2	Three subequal dorsoclinate genal setae	3
–	Middle dorsoclinate genal seta about 1/2 length of setae on either side	4
3	Acrostichal setulae in about 2 rows; surstylus broadly spatulate in lateral view, anteroventral angle very broadly rounded, posteroventral angle relatively broadly projected medially as an acutely pointed process; anterior margin of surstylus bearing distinct row of long setulae (Puerto Rico)	*Paracanace wirthi* Mathis
–	Acrostichal setulae in about 4 rows; surstylus narrow in lateral view, digitiform, slightly angulate; anterior margin of surstylus with few setulae, these not as long as those along posterior margin (Costa Rica: Cocos Islands)	*Paracanace hoguei* Mathis & Wirth
4	Surstylus relatively narrow in lateral view, appearing slipperlike, anterior margin slightly swollen and broadly rounded, tapered ventrally to broadly rounded, ventral margin; posterior margin of surstylus lacking distinct row of longer setulae; posteroventral angle of surstylus noticeably produced apically (widespread in Caribbean)	*Paracanace aicen* Mathis & Wirth
–	Surstylus in lateral view broad on distal 1/2, especially evident in lateral view; ventral, surstylar margin broadly truncate in lateral and posterior views; posterior margin of surstylus bearing distinct row of longer setae (Jamaica)	*Paracanace lebam* Mathis & Wirth
5	Fore- and midfemora of male with row of about 20 long, white setae along proximal ½ of posteroventral margin; surstylus with sub-basal anterior lobe setose and constricted before apical enlargement (Galápagos Islands)	*Paracanace maritima* (Wirth)
–	Fore- and midfemora of male with not more than 10 long, white setae along posteroventral margin at base; surstylus simple, lacking anterior setose lobe or sub-basal constriction	6
6	Tarsi mostly dark, concolorous with tibiae (Galápagos Islands)	*Paracanace cavagnaroi* (Wirth)
–	Tarsi mostly pale, yellowish, especially basitarsomere of hindleg	7
7	Surstylus slender, angulate, length about 3X width (Brazil)	*Paracanace oliveirai* (Wirth)
–	Surstylus broad, truncate ventrally, length not more than twice width, posteroventral angle slightly produced (Panama)	*Paracanace blantoni* (Wirth)

###### Paracanace oliveirai

(Wirth)

http://species-id.net/wiki/Paracanace_oliveirai

Fig. 8

Canace oliveirai Wirth 1956: 164. [Brazil. Rio de Janeiro: Ilha Guaiba, Baia de Sepetiba (22°58.3'S, 43°52.6'W); HT ♂, FIOC]; 1975: 1 [Neotropical catalog].
Paracanace oliveirai . Mathis and Wirth 1978: 524 [generic combination, key], 527 [key]. Mathis 1992: 10 [world catalog]. Munari and Mathis 2010: 24 [world catalog].

####### Diagnosis.

 This species is similar to other species of the *maritima* group but can be distinguished from other congeners by the following combination of characters: As in generic descriptions and key to species with the following details: Generally appearing setulose, although less so than *Paracanace maritima*; body length 1.54–2.05 mm. *Head:* Frons moderately densely golden brown to brownish tan microtomentose; face microtomentose, mostly silvery white, with some faint grayish blue near middle; gena similar in coloration and vestiture to face but more silvery white, with some gray adjacent to anteroventral margin of eye; middle dorsoclinate genal seta subequal in length to setae on either side. *Thorax:* Mesonotum tan to brown, becoming more grayish brown toward lateral margins and posteriorly; acrostichal setulae in 2 rows, posterior pair longer; scutellum gray; pleural area pale gray with some faint bluish coloration. Wing evenly faintly infumate, pale grayish brown; spinelike setulae along costal margin short, length less than half width of 1st costal cell; costal vein ratio 0.13–0.20; M vein ratio 0.37–0.40. Femora and tibiae gray with some darker coloration dorsally; basal 3 tarsomeres yellow, apical 2 yellowish brown to brown; long setae along posteroventral margin of forefemur with apical 1–2 black, others pale. *Abdomen:* Generally gray, dorsum darker, somewhat shiny, with faint metallic reflections, lateral margins dull. Male terminalia (Fig. 8): surstylus pale colored, especially apical half, yellowish orange to pale yellow; surstylus subrectangular in lateral view, oriented posteroventrally, ventral margin broadly and shallowly rounded, not pointed, with posteroventral and anteroventral angles relatively similar, posterior margin in posterior view with shallow swelling subapically, posteroventral portion projected medially, setulae along anterior and posterior margins small and indistinct.

**Figure 7–8.**
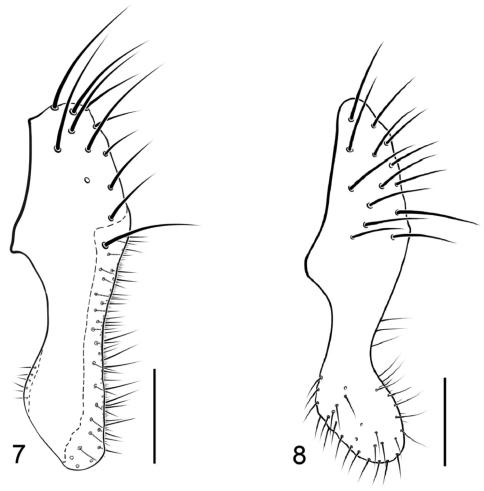
*Paracanace* species **7** *Paracanace aicen*, epandrium, cerci and surstylus, lateral view **8** *Paracanace oliveirai*, epandrium, cerci and surstylus, lateral view

####### Specimens examined from Brazil.

*PARANÁ.* Antonina (25°28.4'S, 48°40.9'W; beach/mangal), 3 Feb-9 Apr 2010, D. and W. N. Mathis (21♂, 4♀; DZUP, USNM); Antonina (25°27.1'S, 48°41.1'W; beach; Ponta da Pita), 3–15 Feb 2010, D. and W. N. Mathis (1♂, 1♀; DZUP, USNM); Prainha (5 km S Matinhos; 25°51.2'S, 48°33.6'W; beach), 15 Nov 2010, D. and W. N. Mathis (1♂; USNM).

*RIO DE JANEIRO.* Ilha da Marambaia (23°3.6'S, 43°59.1'W), 4 Sep 2000, D. and W. N. Mathis (14♂, 6♀; USNM).

####### Distribution.

*Neotropical:* Brazil (Paraná, Rio de Janeiro).

####### Remarks.

 This species is similar and evidently closely related to *Paracanace aicen* Mathis and Wirth from the West Indies, and these two species have been confused. Wirth’s original description and illustration of *Paracanace oliveirai*, for example, included specimens of both species in the type series, and Wirth’s illustration, which is based on a specimen from the Dominican Republic, is actually *Paracanace aicen* (Fig. 7), not *Paracanace oliveirai* (Fig. 8). Because these two species have been confused, we present here comparable lateral views of the respective epandrium, surstylus, and cercus for both species to facilitate their identification. The illustration of *Paracanace oliveirai* is the first for that species. Please note that the lateral view of the fused surstylus of *Paracanace oliveirai* (Fig. 8) is more rectangular than the more elliptical shape of the comparable structure of *Paracanace aicen* (Fig. 7)

##### Genus Procanace

Hendel (30 species worldwide; 1 from Brazil)

http://species-id.net/wiki/Procanace

Procanace Hendel 1913: 93. Type species: *Procanace grisescens* Hendel, by original designation. Mathis 1988: 329–333 [first record of genus from Western Hemisphere]. Munari and Mathis 2010: 25–27 [world catalog].

###### Diagnosis.

 General coloration whitish gray, olivaceous, to blackish brown. *Head:* Interfrontal setae absent, but with a few setulae inserted anteriorly; fronto-orbital setae 3; ocelli arranged to form equilateral or isosceles triangle, if isosceles, the greater distance is between posterior ocelli. Arista pubescent over entire length. Two large dorsoclinate genal setae; anteroclinate genal seta moderately well developed. Palpus not bearing long setae. Epistomal margin, in lateral view, more or less horizontal. *Thorax:* Acrostichal setae, especially a prescutellar pair of large setae, usually lacking (setulae present in species of the *williamsi* group); scutellar disc lacking setae (1–2 pairs of scutellar disc setulae occur in *Procanace nakazatoi* Miyagi of the *williamsi* group); 2 pairs of marginal scutellar setae, apical pair not dorsoclinate; anterior and posterior notopleural setae present, length of both subequal; anepisternum with scattered setulae. Katepisternal seta usually present (lacking in species of the *grisescens* group). Hindtibia lacking spine-like setae apically. *Abdomen:* Male genitalia as follows: Epandrium in posterior view wider than high; cerci reduced, poorly sclerotized; surstylus with an anterior and posterior lobe, the latter larger, sometimes markedly so and shape unique to species.

###### Discussion.

Mathis (1988) first reported the occurrence of *Procanace* in the New World from specimens collected along the tidal shores of the Potomac River in Virginia. This species is now known from coastal habitats on Bermuda and from Virginia south through the West Indies to Brazil. Whether this species is adventive to the New World is unknown but likely.

The only species known from the New World is *Procanace dianneae*, which is in the *cressoni* group of *Procanace* (Mathis 1988). The *cressoni* group is diagnosed by the following combination of external characters: *Head:* Postocellar setae present, subequal to length of ocellar seta; clypeus low, width at least 4X height; palpus yellowish. *Thorax:* Acrostichal setulae lacking; proepisternal seta(e) present; katepisternal seta present.

###### Procanace dianneae

Mathis

http://species-id.net/wiki/Procanace_dianneae

Figs 9–11

Procanace dianneae Mathis 1988: 330 [United States. Virginia. Westmoreland: Westmoreland State Park (banks of Potomac River); figs. of ♂ terminalia; HT ♂; USNM]; 1989: 606–607 [review]; 1992: 11 [world catalog]. Munari and Mathis 2010: 25 [world catalog].

####### Diagnosis.

**Figures 9–11.**
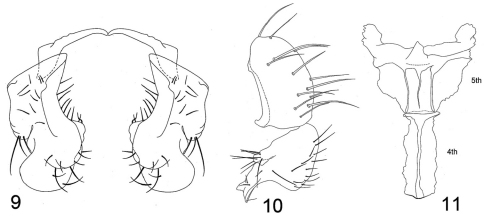
*Procanace dianneae* **9** surstyli, posterior view **10** epandrium and surstylus, lateral view **11** sternites **4** and **5** ventral view.

 Externally this species is very similar to those of the *cressoni* group, and we are tentatively placing it in that group. It differs from the two species of that group, *Procanace cressoni* Wirth and *Procanace taiwanensis* Delfinado, as well as other congeners by the following combination of characters: Moderately small to medium-sized beach flies, body length 2.00–3.10 mm; general coloration whitish gray, olivaceous to brown, scutum darker. *Head:* Postocellar setae well developed, subequal in length to ocellar setae; clypeus low, height 1/4 width; palpus yellowish. *Thorax:* Scutum mostly bluish black, sparsely microtomentose, scutum densely microtomentose, brown; proepisternal seta present, pale; katepisternal seta present; acrostichal setae absent. *Abdomen:* Unicolorous, olivaceous gray with some faint brownish coloration. Male abdomen as follows: Sternite 4 (Fig. 11) narrowly rectangular, over 2X as long as wide; sternite 5 (Fig. 11) wider than long, width of anterior margin subequal to that of sternite 4, becoming wider posteriorly, lateral margins irregular, widest at posterior margin, bearing a short process posterolaterally; epandrium wider than high in posterior view, bearing numerous setae, in lateral view (Fig. 10) posterodorsal margin broadly rounded, ventral margin nearly flat, anterior margin nearly straight except for anteroventral prong and irregular dorsal 1/3; surstylus (Figs 9–10) as 2 processes, anterior one much smaller, digitiform, bearing several setulae preapically and apically, posterior process much larger, length nearly equal to that of epandrium and equally as wide, in lateral view with posterior margin irregularly arched, anteroventral process very angulate in lateral view and spatulate in posterior view.

####### Specimens examined from Brazil.

*PARANÁ.* Antonina (25°27.1'S, 48°41.1'W; beach; Ponta da Pita), 3 Feb 2010, D. and W. N. Mathis (3♂; DZUP, USNM); Antonina (25°28.4'S, 48°40.9'W; beach/mangal), 3 Feb-14 Nov 2010, D. and W. N. Mathis (13♂, 4♀; DZUP, USNM); Paranaguá (Rio Itiberê; 25°31.4'S, 48°30.3'W; 3 m), 23 Jan 2010, D. and W. N. Mathis (4♂, 2♀; DZUP, USNM).

*RIO DE JANEIRO.* Ilha da Marambaia (23°3.6'S, 43°59.1'W), 4 Sep 2000, D. and W. N. Mathis (11♂, 3♀; USNM).

*SÃO PAULO.* Ubatuba, Praia do Estaleiro (23°20.5'S, 44°53'W; beach), 30 Mar 2010, D. and W. N. Mathis (6♂, 1♀; DZUP, USNM).

####### Distribution.

*Nearctic:* Bermuda, United States (Alabama, Delaware, Florida, Maryland, Mississippi, North Carolina, South Carolina, Virginia). *Neotropical:* Brazil (Paraná, Rio de Janeiro, São Paulo), West Indies (Cuba).

####### Natural History.

 All specimens of the type series were collected from the shoreline of the tidal portion of the Potomac River at Westmoreland State Park (Virginia, United States). At the park, the river is over a mile wide, and the water is slightly brackish due largely to the tidal influence. The shore is either almost entirely sandy, the bathing area of the beach, or a combination of sand, considerable gravel, and some cobble and large rocks. In the latter habitat, the shore is quite narrow, at most two to three meters, and immediately adjacent to the shore is a cliff. In the sandy area, specimens occurred along the protected sides of narrow, wooden jetties that were installed perpendicular to the shoreline to break up the action of waves and prevent erosion of the beach. In the sand/cobble/rock habitat, specimens were found only on rocks and were easily collected by sweeping immediately over and between the rocks. Most of the rocks and jetties were covered in part with algae, and we suspect that the larvae of this species were feeding on them.

####### Remarks.

 Much of the temperate and tropical Atlantic Coast of the New World has some of the busiest commercial waterways in the world, and we do not dismiss the possibility that this species was introduced in conjunction with the large volume of traffic on these waters.

This species has a demonstrated ability to disperse well. Although initially discovered in Virginia, where it occurs widely along the state’s maritime coast, the species has now been found from Delaware south to Florida, along the Gulf Coast (Alabama and Mississippi), and into the Neotropics (Cuba and Brazil). The records from the state of Paraná are the southernmost thus far.

#### Subfamily Tethininae

http://species-id.net/wiki/Tethininae

Tethinidae Hendel 1916: 297 [as a family]; 1917: 45. Type genus: *Tethina* Haliday. Mathis and Munari 1996: 1–27 [world catalog]. Munari and Mathis 2010: 40–66 [world catalog].

##### Diagnosis.

 Adult. Small to moderately large flies, body length 1.43–3.66 mm; frequently invested with pale yellowish to brown microtomentum. 3–4 lateroclinate fronto-orbital setae, 3 inclinate frontal setae; postocellar seta convergent. Face sometimes characterized by 2 shiny protuberances laterad of facial cavity above vibrissal pore (*Tethina*); vibrissal seta variable, if present usually weak. 1 proepisternal seta; 1 proepimeral seta (sometimes absent in the genus *Tethina*); anepisternum with 1 or more setae and some setulae posteriorly. Precoxal bridge present. Wing hyaline to infuscate or pale yellow or even patterned (*Tethina pictipennis* Freidberg and Beschovski and *Tethina lusitanica* Munari, Almeida and Andrade); C with Sc break only; cell cup present but small; A_1_ weakened apically, not reaching wing margin. Tibiae lacking preapical dorsal seta. Male epandrium bearing 2 lobes ventrally (the lobe that articulates dorsally with the subepandrial sclerite is considered to be the true surstylus while the anterior surstylar-like lobe may or may not be surstylar in origin); the true surstylus is generally strongly setulose; aedeagal apodeme long, slender; ejaculatory apodeme usually large; aedeagus usually elongate, ribbonlike, sinuous, subcylindrical, with a more or less dense ventral pubescence, often with several microscopic papillae. Female with 2 sclerotized spermathecae; cercus subcylindrical or compressed, sometimes bearing strong, spinelike setulae (pseudacanthophorites); tergites 7–8 mostly with characteristic pigmented areas; epiproct generally small, bearing a pair of setulae dorsally on apical third; hypoproct large.

##### Natural History.

Tethininae are mostly halobiont/thalassophiles, occurring in coastal marine habitats. Adults of thalassophilous species are commonly found in coastal marine habitats (Karl 1930; Munari and Vanin 2007), including the intertidal zone, wrack heaps (usually brown algae that are most abundant along temperate seashores bathed by cold currents), salt marshes, dune vegetation, and on salty soils or bare sand. We have also observed adults often in large numbers on carcasses of marine animals on beaches.

The immature stages of the subfamily are incompletely known. Ferrar (1987) provided some observations on the puparia of *Tethina grisea* (Fallén). Gorczytza (1988) reported on the spatial and seasonal distribution of some European species (*Pelomyiella mallochi* (Sturtevant), *Tethina albosetulosa* (Strobl), *Tethina illota* Haliday, *Tethina flavigenis* (Hendel), and *Tethina grisea* (Fallén)) from a study using color traps on the Frisian Islands of Mellum and Memmert. In nature, an abundance of individuals and a paucity of species sometimes characterize sandy sites where tethinids occur.

##### Key to Genera of Tethininae from Brazil

**Table:** 

1	Eyes densely though minutely setulose. A true vibrissal seta present on vibrissal angle (lacking shiny tubercle above the foremost strong peristomal seta). Male with an anterior surstylarlike lobe in addition to true surstylus, which is fused to epandrium in some species	*Dasyrhicnoessa* Hendel, 1934
–	Eye bare or sparsely setulose. A true vibrissal seta absent but foremost peristomal setae inclinate and simulating vibrissae (the bare vibrissal angle a shiny tubercle above each false vibrissae). Male lacking a surstylarlike lobe but with a true surstylus usually positioned ventrad of epandrium and articulating with it	*Tethina* Haliday, 1837

##### Genus Dasyrhicnoessa

Hendel (25 species worldwide; 1 from Brazil)

http://species-id.net/wiki/Dasyrhicnoessa

Dasyrhicnoessa Hendel 1934:38. Type species: *Rhicnoessa fulva* Hendel, original designation. Malloch 1935:93 [discussion]. Mathis and Munari 1996:11–13 [world catalog]. Munari and Mathis 2010: 43–46 [world catalog].

###### Diagnosis.

*Dasyrhicnoessa* is distinguished from other genera of the family by the following combination of characters: *Head:* Frons bearing some setulae in addition to larger setae; fronto-orbital and orbital setae usually with similar orientation, mostly reclinate or lateroclinate; fronto-orbital setae 3–4; paravertical setae more or less convergent. *Head:* Face lacking shiny tubercle above vibrissal pore; vibrissal seta present on apex of vibrissal angle. Eye mostly densely covered with small, pale, interfacetal setulae. Gena bare except for a ventral or nearly ventral row of setae (peristomal setae); gena narrow, about 1/8–1/3 eye height. Palpus and proboscis usually normally developed; clypeus small, if exposed not protruding anteriad beyond oral margin. *Thorax:* Scutum with numerous rows of coarse setulae arising from punctures; scutellar disc bare; postpronotum with 3 main setae, ventral seta curved upward; acrostichal setulae in two or more complete or nearly complete rows; prescutellar acrostichal setae present; scutellar disc bare except for marginal setae. Wing with costa not spinose; vein A_1_+CuA_2_ short, much shorter than discal cell; wing usually short, about twice as long as wide (less often 2.5–3.0 times); cell bm and discal cell distinct. Forefemur generally bearing an anteroventral ctenidial comb on distal third; mid and hind tibiae evenly setulose, lacking anterodorsal or posterodorsal setae. *Abdomen:* Tergites wider than long; tergite 6 well differentiated from short syntergosternite 7+8, the latter forming a dorsal pregenital sclerite. Male terminalia: Epandrium with a posterior (true) surstylus, articulating with sternite 10. In some species, articulating broadly with ventral margin of epandrium, in others, reduced and positioned more dorsad, along posterior margin of epandrium. Anterior process a surstylarlike lobe, not articulating with sternite 10 but only with anterior margin of epandrium. This lobe much reduced in some species (absent in *Dasyrhicnoessa platypes* Sasakawa) and positioned more or less medially along anterior margin of epandrium. Aedeagus long, sinuous, ribbonlike.

###### Discussion.

 In the New World, a single species, *Dasyrhicnoessa insularis* (Aldrich), is known, and was probably introduced through human commerce. Woodley and Hilburn (1994) and Mathis and Munari (1996) first recorded this genus from the New World (as *Dasyrhicnoessa ferruginea* (Lamb)), and here we provide detailed locality data and descriptive documentation for the genus and the only known species that occurs there. We first discovered the genus and species on barrier islands off the coast of Belize and at the western margin of the Caribbean. Since then, we have found it in the United States (Florida), Mexico (Tabasco), on the Lesser Antilles (Dominica, St. Lucia, St. Vincent), and Bermuda in the western North Atlantic. The genus was probably introduced through human commerce and is now widespread throughout the Caribbean Region and perhaps beyond. Elsewhere, the genus occurs primarily within the Pacific and Indian Ocean basins where 25 species have been described thus far (Munari and Mathis 2010).

*Dasyrhicnoessa* is distinctive and is easily distinguished, especially from other genera of the subfamily Tethininae, by the densely setulose eyes, prominent oral vibrissal seta, vibrissal angle lacking a shiny tubercle, an anterior surstylarlike lobe, and a posterior (true) surstylus in males.

###### Dasyrhicnoessa insularis

(Aldrich)

http://species-id.net/wiki/Dasyrhicnoessa_insularis

Figs 12–14

Tethina insularis Aldrich 1931: 395 [(United States) Wake Island; HT ♀, USNM (41629)].
Rhicnoessa insularis . Hendel 1934: 44 [key], 48 [generic combination, citation].
Dasyrhicnoessa insularis . Hardy and Delfinado 1980: 371–373 [generic combination, citation, figs. of head, wing, ♂ and ♀ terminalia, Oahu, Maui, Hawaii, Frigate Shoal, Pearl and Hermes Reef, Canton Island, and Palmyra Island]. Mathis and Munari 1996: 12 [world catalog]. Munari and Mathis 2010: 44–45 [world catalog].
Tethina lasiophthalma Malloch 1933: 17 [Marquesas. Hivaoa: Tahauku; HT ♂, BPBM]. Munari 1988: 48 [synonymy with *Rhicnoessa ferruginea* Lamb].
Dasyrhicnoessa lasiophthalma . Sasakawa 1974: 2 [generic combination]. Steyskal and Sasakawa 1977: 394 [Oriental catalog]. Foster and Mathis 1998: 606–608 [revision, Caribbean and Gulf of Mexico, figs. of ♂ terminalia]. Munari and Evenhuis 2000: 145 [synonymy].
Dasyrhicnoessa ferruginea of authors, not Lamb 1914 [misidentification]. Woodley and Hilburn 1994: 53 [citation, Bermuda]. Munari and Evenhuis 2000: 145 [citation].
Dasyrhicnoessa freidbergi Munari 1994: 20 [Cameroon. Kribi (beach, Rt. N7); HT ♂, TAU]. Mathis and Munari 1996: 12 [world catalog]. Munari and Evenhuis 2000: 145 [synonymy].

####### Diagnosis.

**Figures 12–14.**
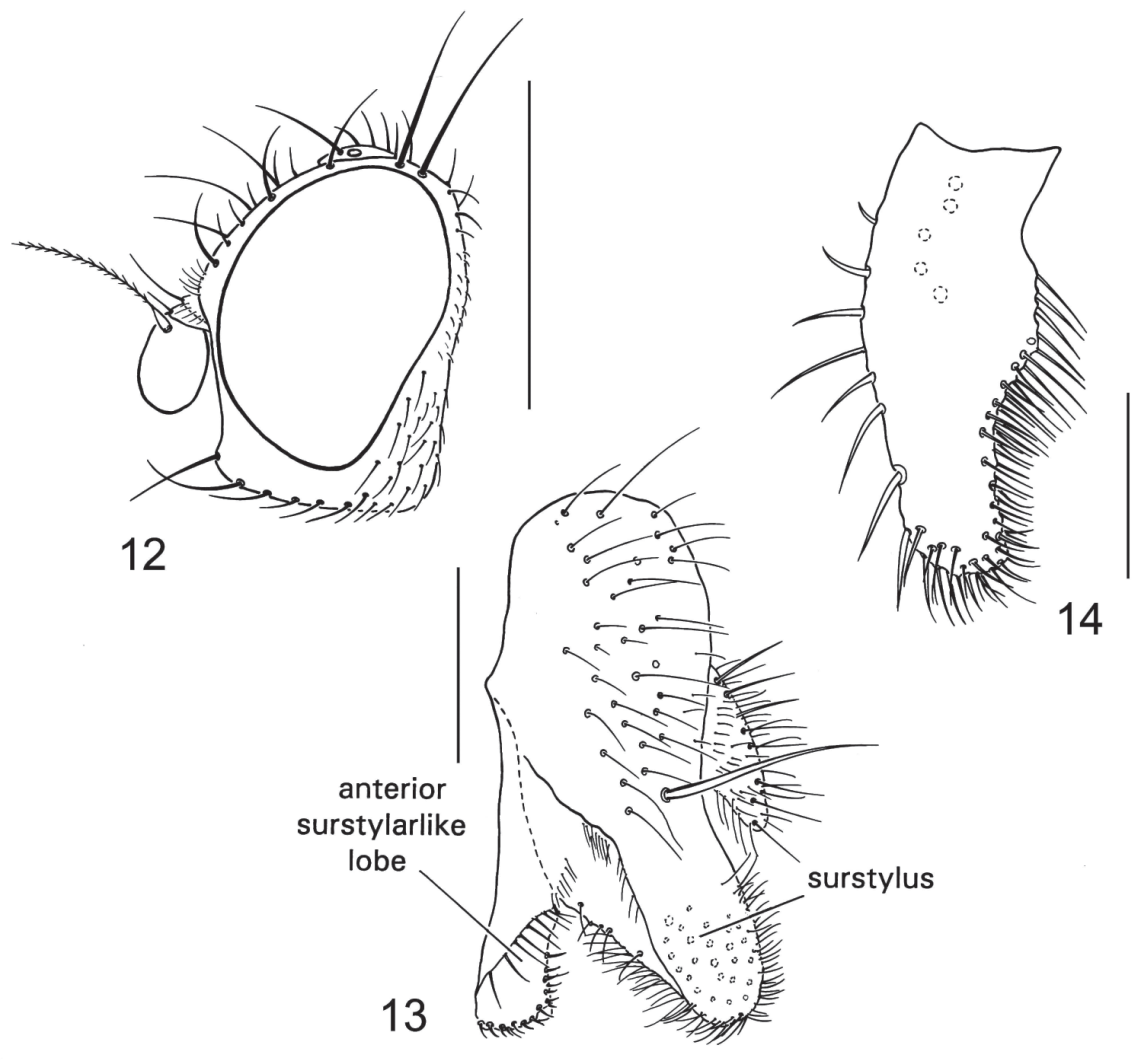
*Dasyrhicnoessa insularis* **12** head, lateral view **13** epandrium, surstylus and anterior surstylarlike lobe, lateral view **14** anterior surstylarlike lobe, posterior view.

 This species is distinguished from congeners by the following combination of characters: *Head* (Fig. 12). *Thorax:* dark orangish brown; acrostichal setulae in 6 rows; legs yellow; forefemur bearing comb of closely set, peglike setae along distal half of anteroventral surface; midfemur bearing ctenidial comb of setae on distal half of posteroventral surface. *Abdomen:* Male terminalia (Figs 13–14): length of anterior surstylar-like lobe equal to or slightly shorter than surstylus; anterior surstylar-like lobe somewhat kidney shaped; surstylus bearing normal to slightly developed setae, none thickly developed.

####### Specimens examined from Brazil.

*PARANÁ.* Antonina (25°28.4'S, 48°40.9'W; beach/mangal), 3 Feb–9 Apr 2010, D. and W. N. Mathis (16♂; DZUP, USNM); Matinhos (N.; 25°46.4'S, 48°30.8'W; 1 m; beach/estuary), 9 Apr 2010, D. and W. N. Mathis (3♂; DZUP, USNM); Paranaguá (Rio Itiberê; 25°31.4'S, 48°30.3'W; 3 m), 23 Jan 2010, D. and W. N. Mathis (5♂, 1♀; DZUP, USNM).

*SÃO PAULO.* Ubatuba, Praia do Estaleiro (23°20.5'S, 44°53'W; beach), 30 Mar 2010, D. and W. N. Mathis (1♂; USNM).

####### Distribution.

*Afrotropical*: Cameroon, Madagascar, Nigeria. *Australasian/Oceanian*: American Samoa (Tutuila), Australia (Queensland), Bismark (Dyaul), Canton Island, Caroline Islands (Ponhpei, Chuuk, Yap, Palau), Fiji Islands (Ovalau, Suva, Viti Levu), ?French Polynesia (Society Islands: Moorea), Hawaii (French Frigate Shoals, Hawaii, Hilo, Lisiansky, Maui, Midway Atoll, Molokai, Oahu, Pearl and Hermes Reef), Kiribati (Butaritari, Makin, Eita, Tarawa, Abemama), Line Islands (Christmas), Mariana Islands (Saipan, Tinian), Marquesas (Hivaoa, Nuku Hiva), Marshall Islands (Majuro, Japtan, Parry, Lib, Jibu, Jaluit, Namorik), Hebrides (Erromanga), Palmyra Island, Pitcairn Island, Rapa Island, Society Islands (Bora Bora), Wake Island. *Nearctic*: Bermuda, United States (Florida). *Neotropical*: Bahamas (South Bimini), Belize, Brazil (Ceará, Paraná, São Paulo), Mexico (Tabasco), West Indies (Cuba, Dominica, St. Kitts, St. Lucia, St. Vincent).

####### Remarks.

 This species was known previously only from the Indo-Pacific area, and its occurrence in the Caribbean, Gulf of Mexico, Bermuda, and now in Brazil represents a significant range extension.

##### Genus Tethina

Haliday (77 species worldwide; 3 from Brazil)

http://species-id.net/wiki/Tethina

Tethina Haliday, in Curtis 1837: 293 (as a subgenus of *Opomyza*; published in synonymy; first made available by use in Haliday 1838: 188). Type species: *Opomyza (Tethina) illota*Haliday 1838, by subsequent monotypy (Haliday 1838: 188). Sturtevant 1923: 5–7 [discussion of synonymy, listing of Nearctic species]. Thompson and Mathis 1981: 86 [citation, nomenclature]. Mathis and Munari 1996: 13–19 [world catalog]. Foster and Mathis 1998: 608–630 [revision of Caribbean and Gulf of Mexico species]. Sabrosky 1999: 32, 304 [citations, nomenclature]. Munari and Mathis 2010: 48–66 [world catalog].
Rhicnoessa Loew 1862: 174. Type species: *Rhicnoessa cinerea* Loew, by monotypy. Loew 1865: 34–39 [revision]. Williston 1908: 292, 296 [fig. of head, key]. Collin 1911: 234 [probable synonymy with *Tethina*]. Malloch 1913: 147 [discussion, fig. of head]. Hendel 1917: 46 [synonymy in key]; 1934: 46 [references]. Munari 1990: 60–61 [status as a subgenus of *Tethina*].
Phycomyza Melander 1952: 198. Type species: *Rhicnoessa milichioides* Melander, by original designation. Vockeroth 1965: 727 [Nearctic catalog]. Foster 1976a: 338 [synonymy].

###### Diagnosis.

*Tethina* is distinguished from other genera of the subfamily Tethininae by the following combination of characters: *Head:* Frons bearing some setulae in addition to larger setae; fronto-orbital and orbital setae usually with similar orientation, mostly reclinate or lateroclinate; fronto-orbital setae 3–4; postocellar setae more or less convergent (lacking in *Tethina lusitanica*). Face with shiny tubercle above vibrissal pore. Eye appearing bare, setulae very sparse or lacking. Gena bare (except for *Tethina pictipennis* and *Tethina lusitanica*, which have scattered, inconspicuous setulae) except for a ventral or nearly ventral row of setulae; gena high in many species, height 0.50–0.75 that of eye height. Palpus and proboscis usually normally developed; clypeus small, if exposed not protruding anteriad beyond oral margin. *Thorax:* Scutum generally with more or less numerous rows of coarse setulae arising from punctures; scutellar disc bare; postpronotum with 3 or more setae, ventral seta curved upward; acrostichal setulae in two or more complete or nearly complete rows (lacking in *Tethina lusitanica*); prescutellar acrostichal setae present (lacking in *Tethina lusitanica*). Wing with costa not spinose; vein A_1_+CuA_2_ short, much shorter than discal cell; wing usually shorter, about twice as long as wide (less often 2.5–3.0 times); cell bm and discal cell distinct. Mid and hind tibiae evenly setulose, lacking anterodorsal or posterodorsal setae. *Abdomen:* Tergites wider than long; tergite 6 well differentiated from short syntergosternite 7+8, the latter forming a dorsal pregenital sclerite. Male terminalia: Surstylus positioned at ventral margin of epandrium, usually broadly articulated externally with epandrium, internally with subepandrial sclerite; aedeagus usually very long and sinuous, either thick and straplike or narrow and ribbonlike; aedeagus micropubescent dorsally.

###### Discussion.

 Worldwide among genera of Tethininae, *Tethina* has more than half of the described species (77 of 115) (Munari 2002). Two species occur in the study area and a third, *Tethina albula* (Loew), has been reported (Prado and Tavares 1966) but not seen as part of this study. Since *Tethina albula* has been reported from Brazil, and as there is the possibility of its occurrence there, we have included it in the key to species. The included species of *Tethina* occur along maritime beaches of the littoral biotic region. Specimens are sometimes abundant, especially on fresh and decomposing wrack.

###### Key to Species of Tethina from Brazil

**Table:** 

1	Gena short, 0.33 or less height of eye; setae and setulae black. Apex of scutellum with yellowish to reddish spot (may vary in size but always obvious)	*Tethina xanthopoda* (Williston)
–	Gena high, 0.37–0.75 height of eye; setae and setulae mostly white. Apex of scutellum uniformly gray microtomentose	2
2	Surstylus in lateral view straight	*Tethina willistoni* (Melander)
–	Surstylus in lateral view curved anteroventrally	*Tethina albula* (Loew)

###### Tethina willistoni

Melander

http://species-id.net/wiki/Tethina_willistoni

Figs 15–17

Anthomyza cinerea Williston 1896: 444 [West Indies. St. Vincent. Wallilabou beach (13°15'N, 61°16'W); NT ♂ (designated by Foster and Mathis 1998: 615), USNM; preoccupied, Loew 1862].
Rhicnoessa cinerea . Czerny 1902: 256 [generic combination].
Rhicnoessa willistoni Melander 1913: 298 [new name for *Anthomyza cinerea* of Williston 1896, not Loew 1862]. Hendel 1934: 51 [citation]. Melander 1952: 201 209 [key, citation].
Tethina willistoni . Foster 1976b: 3 [generic combination, Neotropical catalog]. Mathis and Munari 1996: 19 [world catalog]. Foster and Mathis 1998: 611, 613, 615–618 [revision, Caribbean and Gulf of Mexico, neotype designation, figs. head and ♂ terminalia]. Munari and Mathis 2010: 65 [world catalog].
Rhicnoessa bermudaensis Melander 1952: 203 [Bermuda. Castle and Cooper Islands; LT ♂ (designated by Foster and Mathis 1998: 612), USNM]. Mathis and Foster 2007: 421 [synonymy].
Tethina bermudaensis . Vockeroth 1965: 727 [generic combination, Nearctic catalog]. Woodley and Hilburn 1994: 53–54 [citation, Bermuda]. Mathis and Munari 1996: 15 [world catalog]. Foster and Mathis 1998: 611–613 [revision, lectotype designation, Caribbean and Gulf of Mexico, fig. of ♂ terminalia].
Rhicnoessa variseta Melander 1952: 209 [United States. California. Orange: Corona del Mar; LT ♂ (designated by Foster and Mathis 1998: 616), USNM]. Foster and Mathis 1998: 615 [synonymy, lectotype designation].
Tethina variseta . Vockeroth 1965: 728 [generic combination, Nearctic catalog]. Mathis and Munari 1996: 19 [world catalog].
Tethina carioca Prado and Tavares 1966: 433 [Brazil. Rio de Janeiro: Ilha do Governador (Galeão); HT ♂, FIOC (13356); figs. of ♂ terminalia and wing]. Foster 1976b: 2 [Neotropical catalog]. Mathis and Munari 1996: 15 [world catalog]. Foster and Mathis 1998: 615 [synonymy].
Tethina albula of authors, not Loew 1869 [misidentification]. Frey 1919: 15.

####### Diagnosis.

**Figures 15–17.**
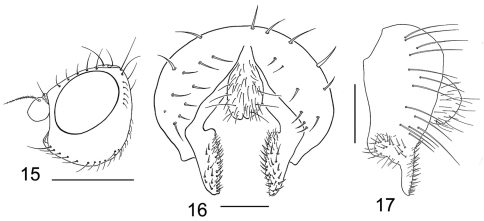
*Tethina willistoni* **15** head, lateral view **16** epandrium, cerci and surstylus, posterior view **17** same, lateral view.

 This species is distinguished from congeners by the following combination of characters: Body length 1.65–3.00 mm; body generally whitish gray to gray, microtomentose; setae generally white to slightly off white but sometimes with all setae black. *Head* (Fig. 15): Gena high, greater than 0.5 eye height. *Thorax:* 4 irregular rows of acrostichal setulae; scutellum uniformly gray; femora mostly yellow to mostly gray; hindfemora of male similar to or only slightly more swollen than fore- and midfemora; tibiae yellow; basal 4 tarsomeres yellow, apical tarsomere brown. *Abdomen:* Male terminalia (Figs 16–17): Surstylus articulated with and broadly attached to epandrium, broadly spatulate/triangular in posterior view, length 2–3× width, apex broadly rounded; medial margin bearing numerous short, stout setulae along entire length; surstylus in lateral view narrow, tapered to apical point, posterior margin almost straight; basal portion produced anteriorly as a broadly rounded lateral lobe bearing several short setulae medially; aedeagus thick, straplike.

####### Specimens examined from Brazil.

*PARANÁ.* Matinhos (N.; 25°46.4'S, 48°30.8'W; 1 m; beach/estuary), 25 Mar-9 Apr 2010, D. and W. N. Mathis (6♂; DZUP, USNM); Paranaguá (Rio Itiberê; 25°31.4'S, 48°30.3'W; 3 m), 23 Jan 2010, D. and W. N. Mathis (8♂, 1♀; DZUP, USNM).

*RIO DE JANEIRO.* Ilha do Governador (Galeão; 22°47.8'S, 43°14.7'W), 11 Oct 1966, A. P. do Prado and Tavares (1♂; MZUSP).

*SANTA CATARINA.* Barra Velha (26°38'S, 48°40.9'W; beach), 29 Apr 2010, D. and W. N. Mathis (8♂, 1♀; DZUP, USNM).

*SÃO PAULO.* Ubatuba, Praia Puruba (23°21'S, 44°55.6'W; beach), 29 Mar 2010, D. and W. N. Mathis (6♂, 2♀; DZUP, USNM); Ubatuba, Praia do Estaleiro (23°20.5'S, 44°53'W; beach), 30 Mar 2010, D. and W. N. Mathis (1♂; USNM).

####### Distribution.

*Australasian/Oceanian:*Hawaii (French Frigate Shoals, Hawaii, Kahoolawe, Kauai, Lisiansky, Maui, Oahu), Midway Islands. *Nearctic:*Bermuda, United States (California, Connecticut, Delaware, Florida, Maryland, Massachusetts, North Carolina, South Carolina, Virginia). *Neotropical*: Bahamas, Belize, Brazil (Paraná, Rio de Janeiro, Santa Catarina, São Paulo), Cuba, Curaçao, Ecuador, Mexico (Chihuahua, Tabasco), Panama, Peru, Tobago, Turks and Caicos, West Indies (Anguilla, Antigua, Barbados, Barbuda, Dominica, Dominican Republic, Grand Cayman, Grenada, Jamaica, Montserrat, Puerto Rico, St. Croix, St. Lucia, St. Vincent).

####### Remarks.

 Some slight variation was evident in the shape of the surstyli within specimens of this species. In posterior view the surstylus varies from being shorter and more exactly triangular to being slightly longer but still triangular. Previously we considered these differences to represent separate species, *Tethina bermudaensis* and *Tethina willistoni*. After examination of many dissected specimens from Canada south through southern Brazil, we agree with Foster and Mathis (2008) that this variation is intraspecific.

The variation in setal coloration and size of *Tethina willistoni* is remarkable. The variation in external characters is as follows: the more robust specimens from the Carribean areas have mostly stout, black setae and often present a very “bristly” habitus (similar to *Tethina spinulosa* and *Tethina horripilans*). Smaller, more delicate specimens have only the apical scutellar setae black with all other setae being white. Many specimens fall between these two extremes, making it virtually impossible to distinguish between *Tethina willistoni* and other species on the basis of external structures alone. A very similar chaetochromatic variation is also found in the Western Palearctic *Tethina albosetulosa* (Strobl) (Munari and Canzoneri 1992; Munari and Vanin 2007).

###### Tethina xanthopoda

(Williston)

http://species-id.net/wiki/Tethina_xanthopoda

Figs 18–20

Anthomyza xanthopoda Williston 1896: 445 [West Indies. St. Vincent; LT ♂ (designated by Foster and Mathis 1998: 620); BMNH]. Czerny 1902: 256 [citation, placement in *Rhicnoessa*].
Tethina xanthopoda . Foster 1976b: 3 [generic combination, Neotropical catalog]. Woodley and Hilburn 1994: 54 [citation, Bermuda]. Mathis and Munari 1996: 19 [world catalog]. Foster and Mathis 1998: 620–624 [revision, Caribbean and Gulf of Mexico, lectotype designation, figs. of head and ♂ terminalia]. Munari and Mathis 2010: 66 [world catalog].
Rhicnoessa xanthopoda . Czerny 1902: 256 [generic combination]. Melander 1913: 298 [key]; 1952: 202 209 [key, citation]. Hendel 1934: 51 [citation].
Rhicnoessa seriata Melander 1952: 206 [United States. Florida. Dade: Miami; LT ♂ (designated by Foster and Mathis 1998: 620), USNM]. Foster and Mathis 1998: 620 [synonymy, lectotype designation].
Tethina seriata . Vockeroth 1965: 728 [generic combination, Nearctic catalog]. Mathis and Munari 1996: 18 [world catalog].
Tethina brasiliensis Prado and Tavares 1966: 435 [Brazil. Rio de Janeiro: Ilha do Governador (Galeão); HT ♂, FIOC (13358); figs. of ♂ and ♀ terminalia]. Foster 1976b: 2 [Neotropical catalog]. Artigas et al. 1992: 127–129 [figs. of puparium]. Mathis and Munari 1996: 15 [world catalog]. Foster and Mathis 1998: 620 [synonymy].

####### Diagnosis.

**Figures 18–20.**
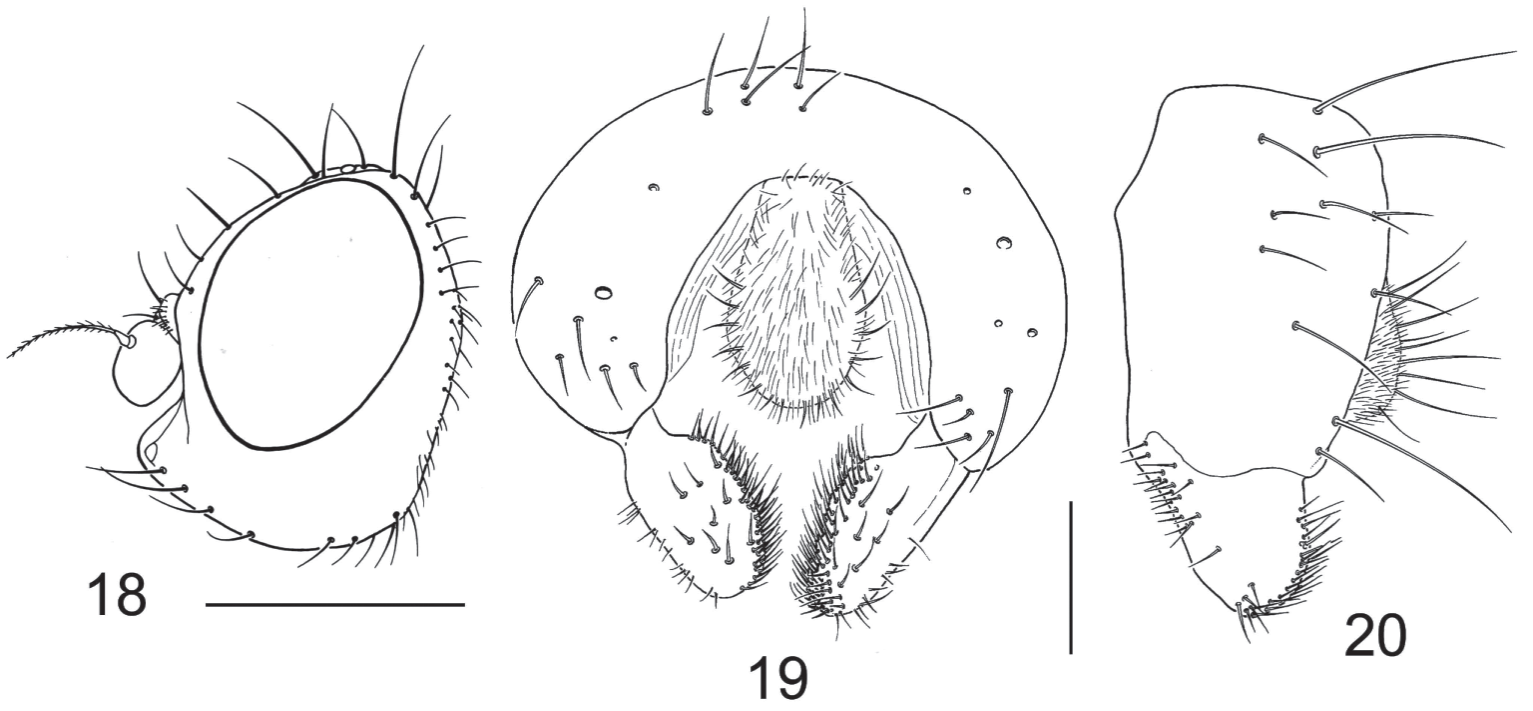
*Tethina xanthopoda* **18** head, lateral view **19** epandrium, cerci and surstylus, posterior view **20** same, lateral view.

 This species is distinguished from congeners by the following combination of characters: Body length 1.70–3.10 mm; body with gray microtomentum; setae generally black. *Head* (Fig. 18): Gena short, less than 0.5 eye height. *Thorax:* 4 somewhat irregular rows of acrostichal setulae; apex of scutellum with yellowish to reddish spot (sometimes variable in size but always obvious); femora yellow; hindfemora of male similar to or only slightly more swollen than fore- and midfemora; tibiae and basal 4 tarsomeres yellow, apical tarsomere brown. *Abdomen:* Male terminalia (Figs 19–20): surstylus articulated with and broadly attached to epandrium, broadly spatulate in posterior view, length less than twice width, median margin bearing dense patch of robust setulae along entire length, apex broadly rounded; surstylus in lateral view broadly developed, lateral margin only slightly narrowed posteriorly, apex broadly rounded, lateral surface mostly bare, basal portion only slightly produced anteriorly, bearing moderately dense patch of setulae; aedeagus narrow, ribbonlike.

####### Specimens examined from Brazil.

*RIO DE JANEIRO.* Ilha do Governador (22°47.8'S, 43°14.7'W), Nov 1963, H. Souza Lopes (1♂; MZUSP).

*SANTA CATARINA.* Barra Velha (26°38'S, 48°40.9'W; beach), 29 Apr 2010, D. and W. N. Mathis (12♂; DZUP, USNM).

*SÃO PAULO.* Ubatuba, Praia do Estaleiro (23°20.5'S, 44°53'W; beach), 30 Mar 2010, D. and W. N. Mathis (3♂; USNM).

####### Distribution.

*Nearctic*: Bermuda, Canada (Alberta), United States (Florida). *Neotropical*:Bahamas, Belize, Brazil (Bahia, Rio de Janeiro, Rio Grande do Norte, Santa Catarina, São Paulo), Guyana, Mexico (Quintana Roo, Yucatan), Panama, Trinidad and Tobago, Turks and Caicos, West Indies (Antigua, Barbados, Barbuda, Cuba, Curaçao, Dominica, Dominican Republic, Grand Cayman, Grenada, Jamaica, St. Lucia, St. Vincent).

####### Remarks.

 This widespread species can easily be distinguished from *Tethina cohiba* (often collected at the same locality) in having an obvious reddish yellow spot on the apex of the scutellum. Some specimens must be examined with the scutellum oriented to be directly viewed from behind and with good lighting. In most specimens, however, the spot is immediately obvious. Additional external characters include the mostly yellow femora, which are moderately swollen, as in *Tethina cohiba*.

## Supplementary Material

XML Treatment for Canacidae
